# Detection and evaluation of myocardial fibrosis in Eisenmenger syndrome using cardiovascular magnetic resonance late gadolinium enhancement and T1 mapping

**DOI:** 10.1186/s12968-022-00880-2

**Published:** 2022-11-21

**Authors:** Chao Gong, Jinghua Guo, Ke Wan, Lili Wang, Xiaolin Chen, Jiajun Guo, Juan He, Lidan Yin, Bi Wen, Shoufang Pu, Chen Chen, Yucheng Chen

**Affiliations:** 1 Cardiology Division, Department of Cardiology, West China Hospital, Sichuan University, Guoxue Xiang No. 37, Chengdu, Sichuan Province 610041 People’s Republic of China; 2 Department of Cardiology, The Second People’s Hospital of Yibin, Yibin, Sichuan 610041 People’s Republic of China; 3 Department of Geriatrics, West China Hospital, Sichuan University, Chengdu, Sichuan 610041 People’s Republic of China

**Keywords:** Eisenmenger syndrome, Cardiovascular magnetic resonance, Myocardial fibrosis, T1 mapping, Extracellular volume

## Abstract

**Background:**

Myocardial fibrosis is a common pathophysiological process involved in many cardiovascular diseases. However, limited prior studies suggested no association between focal myocardial fibrosis detected by cardiovascular magnetic resonance (CMR) late gadolinium enhancement (LGE) and disease severity in Eisenmenger syndrome (ES). This study aimed to explore potential associations between myocardial fibrosis evaluated by the CMR LGE and T1 mapping and risk stratification profiles including exercise tolerance, serum biomarkers, hemodynamics, and right ventricular (RV) function in these patients.

**Methods:**

Forty-five adults with ES and 30 healthy subjects were included. All subjects underwent a contrast-enhanced 3T CMR. Focal replacement fibrosis was visualized on LGE images. The locations of LGE were recorded. After excluding LGE in ventricular insertion point (VIP), ES patients were divided into myocardial LGE-positive (LGE^+^) and LGE-negative (LGE^−^) subgroups. Regions of interest in the septal myocardium were manually contoured in the T1 mapping images to determine the diffuse myocardial fibrosis. The relationships between myocardial fibrosis and 6-min walk test (6MWT), N-terminal pro-brain natriuretic peptide (NT-pro BNP), hematocrit, mean pulmonary arterial pressure (mPAP), pulmonary vascular resistance index (PVRI), RV/left ventricular end-systolic volume (RV/LV ESV), RV ejection fraction (RVEF), and risk stratification were analyzed.

**Results:**

Myocardial LGE (excluding VIP) was common in ES (16/45, 35.6%), and often located in the septum (12/45, 26.7%). The clinical characteristics, hemodynamics, CMR morphology and function, and extracellular volume fraction (ECV) were similar in the LGE^+^ and LGE^−^ groups (all *P* > 0.05). ECV was significantly higher in ES patients (28.6 ± 5.9% vs. 25.6 ± 2.2%, *P* < 0.05) and those with LGE^−^ ES (28.3 ± 5.9% vs. 25.6 ± 2.2%, *P* < 0.05) than healthy controls. We found significant correlations between ECV and log NT-pro BNP, hematocrit, mPAP, PVRI, RV/LV ESV, and RVEF (all *P* < 0.05), and correlations trends between ECV and 6MWT (*P* = 0.06) in ES patients. An ECV threshold of 29.0% performed well in differentiating patients with high-risk ES from those with intermediate or low risk (area under curve 0.857, *P* < 0.001).

**Conclusions:**

Myocardial fibrosis is a common feature of ES. ECV may serve as an important imaging marker for ES disease severity.

**Supplementary Information:**

The online version contains supplementary material available at 10.1186/s12968-022-00880-2.

## Background

As the extreme end of the uncorrected congenital heart disease-related pulmonary artery hypertension spectrum, Eisenmenger syndrome (ES) is characterized by cyanosis, secondary erythrocytosis, and multi-organ disorder and is associated with significant morbidity and mortality [1, 2]. Ventricular dysfunction is one of the risk indicators for poor prognosis; nonetheless, the related mechanisms were not well defined [2–4]. Myocardial fibrosis is a common pathophysiological process involved in many cardiovascular diseases. The chronic cyanosis in ES could trigger myofibroblast activity by various signaling pathways, leading to myocardial fibrosis [5–7]. A pathological autopsy study has demonstrated the existence of ventricular myocardial fibrosis in ES [8]. However, the clinical relevance of myocardial fibrosis in ES patients has not been clarified.

Myocardial fibrosis includes focal replacement and diffuse interstitial fibrosis that could be detected, respectively, by the late gadolinium enhancement (LGE) and T1 mapping techniques of cardiovascular magnetic resonance (CMR) [9–11]. Broberg et al. suggested that LGE was prevalent in ES yet not correlated with disease severity, ventricular remodeling, or survival [12]. Several studies suggested that diffuse myocardial fibrosis was present in pulmonary hypertension (PH) [13–18]. Furthermore, our recent study suggested that shunt location was significant for changes in diffuse myocardial fibrosis in ES patients [19]. However, the clinical significance of myocardial fibrosis evaluated by CMR LGE and T1 mapping in ES patients remains largely unexplored. Therefore, this study aimed to investigate potential associations between myocardial fibrosis evaluated by the CMR LGE and T1 and risk stratification profiles including clinical severity, pulmonary artery hemodynamics, and right ventricular (RV) function in ES patients.

## Methods

### Study population

This prospective observational study was registered with the Chinese Clinical Trials Registry (ChiCTR1800019314). From January 2013 to December 2019, consecutive adult patients in our hospital with a definite diagnosis of ES were prospectively enrolled. ES diagnosis was established according to the following criteria: (1) known intra-cardiac or extra-cardiac non-restrictive congenital defect at the atrial, ventricular, or arterial level (isolated or a combination of defects); (2) reversed or bidirectional shunt resulting in hypoxemia (cutaneous oxygen saturation < 92% at rest or < 87% with exercise [20–22]); (3) hemodynamic measurements of resting mean pulmonary arterial pressure (mPAP) ≥ 25 mmHg, pulmonary artery wedge pressure (PAWP) ≤ 15 mmHg, and pulmonary vascular resistance (PVR) > 3 Wood units. Patients with complex congenital heart disease types (e.g., transposition of the great arteries, double-outlet RV, univentricular heart) or with a relatively small defect (atrial septal defect (ASD) < 2.0 cm or ventricular septal defect (VSD) < 1.0 cm) [23]. Patients with a contraindications for CMR, including a history of contrast allergy, implantation of implantable cardioverter defibrillator or pacemaker, or claustrophobia were excluded.

In total, 54 patients met the clinical diagnostic criteria for adult ES and underwent CMR examination. Furtherly, we excluded 3 patients with ES and atrial fibrillation whose cine images could not analyze cine data including RV volume and RV ejection fraction (RVEF), and additionally 6 ES patients who had suboptimal T1 data quality. Ultimately, a total of 45 ES patients were included in the present study. Among them, there were 11 patients (24.4%) of simple ASD, 2 patients (4.4%) of anomalous pulmonary venous drainage (APVD) complicated with ASD, 1 patient (2.2%) of aortopulmonary window, 17 patients (37.8%) of VSD, 9 patients (20.0%) of patent ductus arteriosus (PDA), 3 patients (6.7%) of combined VSD and PDA, 1 patient (2.2%) of atrioventricular septal defect, and 1 patient (2.2%) of combined ASD and PDA. With the exception for isolated extra-cardiac defects (aortopulmonary window or PDA) and pre-tricuspid shunts (ASD or APVD complicated with ASD), a total of 35 patients (77.8%) with intra-cardiac defects and 32 patients (71.1%) with post-tricuspid shunts were included in the subgroup analysis.

The study also included a control group of 30 individuals with a similar age and sex distributions to the ES group who underwent contrast-enhanced CMR examination and selected from our database of healthy volunteers [24]. The study followed the tenets of the Declaration of Helsinki. All participants gave written informed consent, and the local institutional ethics committee at West China Hospital approved the study protocol (2018271).

### Clinical assessment

All patients were clinically and hemodynamically stable at study enrollment. Medical history was recorded, and a physical examination was performed. All patients performed a non-encouraged 6-min walk test (6MWT), and the distance was recorded [25]. World Health Organization (WHO) functional class was evaluated for each patient. Plasma N-terminal pro-brain natriuretic peptide (NT-pro BNP) and hematocrit (Hct) levels were collected from the hospital electronic medical record system. Peripheral arterial oxygen saturation (SpO_2_%) at rest and the end of the 6MWT were recorded in a pre-defined form. The risk stratification profiles of the ES patients were evaluated according to the 6th World Symposium on Pulmonary Hypertension strategy proposed by Galie et al. [26]. Low-, intermediate-, and high-risk strata were defined by estimated 1-year mortality risks of < 5%, 5–10%, and > 10%, respectively. The specific risk strategy approach can be found in Additional file 1. The time difference between the clinical assessment and the CMR acquisitions was ≤ 7 days.

### CMR protocol

CMR acquisitions were performed using a 3 T scanner (Magnetom Tim Trio or Skyra; Siemens Healthineers, Erlangen, Germany) using a dedicated 32-channel phase array cardiac coil or a 30-channel body coil with simultaneous electrocardiographic (ECG) gating technique within 72 h after right heart catheterization (RHC) examination. Among the ES participants, 22 (48.9%) were examined by the Trio/32-channel phase array cardiac coil, and the rest by the Skyra/30-channel body coil. Cine imaging was acquired using an ECG-gated balanced steady-state free precession (bSSFP) sequence during end-expiratory breath-hold in standard short-axis cine views and 2-, 3-, and 4-chamber longitudinal views, covering both ventricles from the basal to the apical level. The specific sequence parameters were as follows: echo time (TE), 1.3 ms; repetition time (TR), 3.4 ms; temporal resolution, 35–45 ms; flip angle (FA), 50°; matrix size, 256 × 192; field-of-view (FOV), 340 × 320 mm; spatial resolution, 1.5 × 1.3 mm; slice thickness, 8 mm without gaps. Gadopentetate dimeglumine (Magnevist; Bayer Healthcare, Berlin, Germany) was administered intravenously at 0.15 mmol/kg. Short- and long-axis LGE images were obtained using the inversion recovery turbo fast low-angle shot sequence with phase-sensitive reconstruction 10–15 min after contrast injection at slice locations identical to those acquired by cine CMR. The scanning time per slice usually ranged from 8 to 12 s, depending on the patient’s heart rate. The LGE parameters were TR, 700 ms; TE, 1.56 ms; flip angle, 20°; matrix size, 256 × 192; slice thickness, 8 mm; FOV, 340 × 320 mm; acquired and reconstructed voxel size, 1.8 × 1.4 × 8 mm.

T1 mapping images were scanned using the motion-corrected modified Look-Locker inversion recovery (MOLLI) sequence before and after contrast injection in three short-axis slices (basal, mid, and apical) with a fixed 5(3)3/4(1)3(1)2 scheme, respectively. Post-contrast T1 measurements were performed approximately 15 min after contrast injection. The parameters for MOLLI included non-selective inversion pulse; bSSFP single-shot readout with a 35° flip angle; minimum inversion time (TI), 110 ms; TI increment, 80 ms; TR/TE, 2.9/1.12 ms; FOV, 360 × 272 mm; matrix size, 256 × 192; acquired and reconstructed voxel size, 2.1 × 1.4 × 8 mm; slice thickness, 8 mm. LGE and T1 mapping sequences were acquired during the end-diastolic cardiac phase.

### Image analysis

Cardiac volumes and mass were determined by manually drawing the end-diastole and end-systole endocardial and epicardial borders using the Qmass software (version 8.1, Medis Medical Imaging, Leiden, the Netherlands) and indexed to the body surface area following the Society for Cardiovascular Magnetic Resonance post-processing guidelines [27]. Papillary muscles and trabeculations were considered part of the cavity volume and excluded from the myocardial mass. LGE images were qualitatively assessed for myocardium hyperintensity. LGE was determined when focal myocardial enhancement was visible in either short- or long-axis view by two independent observers (CG and LLW, both with three years of CMR experience) blinded to the clinical data. Disagreements were resolved by consensus or through consultation with a third reviewer (YCC with over ten years of CMR experience). The location of each LGE region was recorded and classified into ventricular insertion point (VIP), septum, RV myocardium, RV trabeculae, left ventricular (LV) myocardium, or LV papillary. After excluding LGE in VIP, patients with ES were divided into myocardial LGE-positive (LGE^+^) and LGE-negative (LGE^−^) subgroups.

**Fig. 1:**
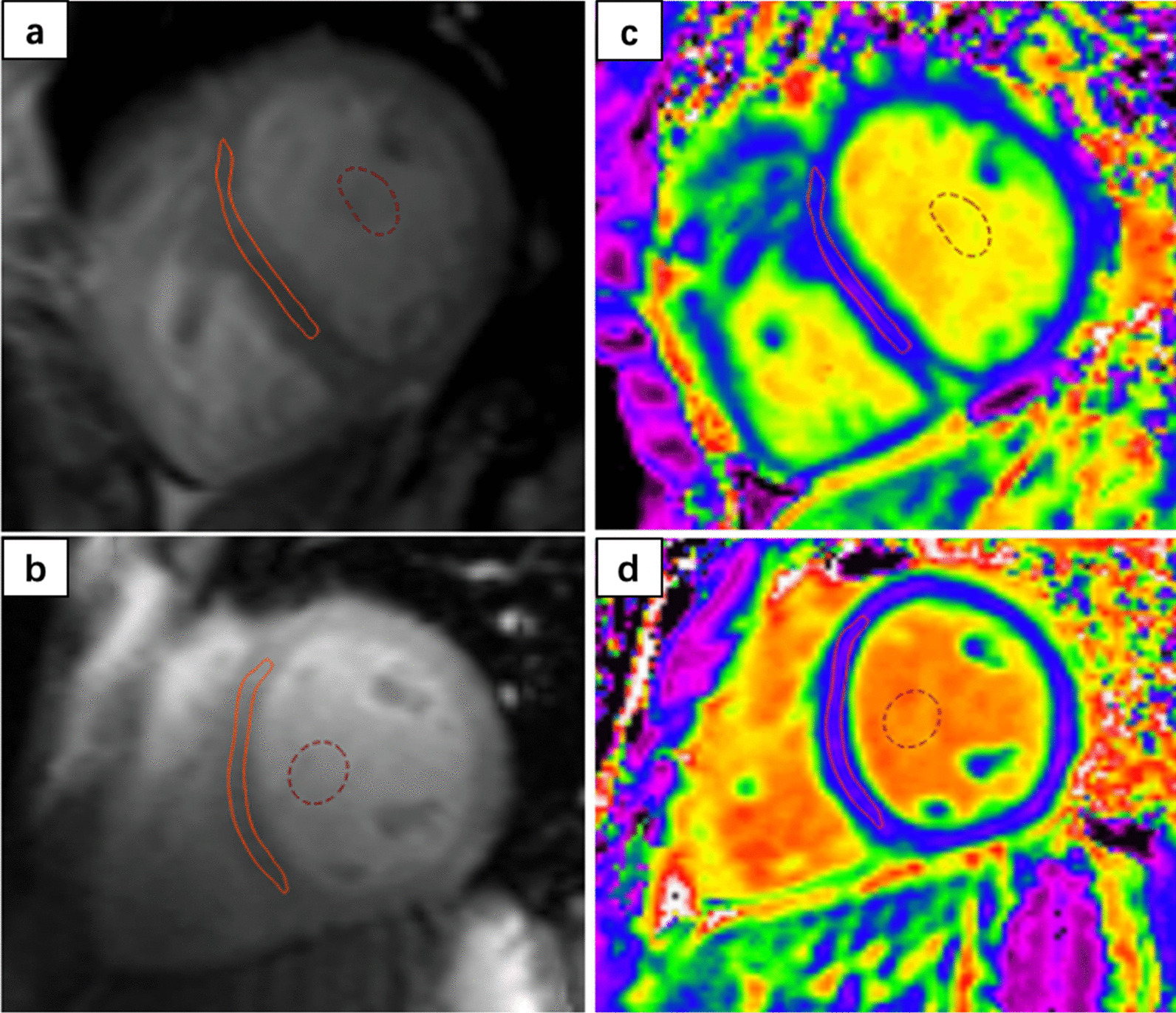
Representative ROI contours on native T1 and extracellular volume fraction (ECV) mapping images of a normal control (**a** native T1 mapping; **c** ECV mapping) and a patient with Eisenmenger syndrome (ES) (**b** native T1 mapping; **d** ECV mapping). The orange solid contour line represents the septum. The red dotted contour represents the blood pool. *ROI* region of interest, *ECV* extracellular volume, *ES* Eisenmenger syndrome

Pre- and post-T1 mapping images were analyzed on the mid-ventricular LV short-axis slice with dedicated QMap software (version 2.2.6, Medis Medical Imaging). The largest possible regions of interest (ROI) for septal myocardium sampling were manually contoured, strictly excluding any LGE (Fig. 1). Furthermore, epicardial fat, trabeculations, and near-wall blood were carefully excluded from the ROI. A T1 value of the blood pool was acquired by drawing an ROI in the LV cavity. The extracellular volume fraction (ECV) was calculated using the following formula: ECV = (1 − Hct) × ([1/T1myocardium post–1/T1 myocardium pre]/[1/T1blood post–1/T1blood pre]). The Hct was measured within 24 h of the CMR scanning.

### Right heart catheterization

All patients underwent RHC to confirm the diagnosis and evaluate the disease severity. RHC was performed routinely in free-breathing patients following a standard procedure [23]. The following invasive hemodynamic measurements were attained: mean right atrial pressure (mRAP), mPAP, PAWP, and the pulmonary-to-systemic flow ratio (Qp:Qs). Cardiac output (CO) was calculated by the indirect Fick equation. PVR was calculated using the formula: PVR = (mPAP − PAWP/CO).

### Statistical analysis

Continuous data are expressed as mean ± standard deviation if the Kolmogorov–Smirnov test showed normal distribution; otherwise, data are presented as the median and interquartile range. Categorical variables are presented as frequency and percentage. Independent Student’s *t*-test and the Mann–Whitney *U* test compared continuous variables with normal and skewed distributions, respectively. Fisher’s exact or χ^2^ test compared categorical variable groups. Associations between variables were tested using Pearson’s correlation coefficients. Receiver operating characteristic (ROC) curve analysis was used to identify the largest area under the curve (AUC) for discriminating high-risk from low- and intermediate-risk profiles in patients with ES. Statistical analysis was performed using SPSS (version 23.0, Statistical Package for the Social Sciences, International Business Machines, Inc., Corp., Armonk, New York, USA) and GraphPad Prism for Macintosh, Version 7.0 (GraphPad Software, San Diego, California, USA). For all tests, a two-tailed *P*-value < 0.05 was considered statistically significant.

## Results

### Baseline characteristics

Overall, 45 ES patients (female, 32/45, 71.1%; 36.6 ± 11.1 years) and 30 healthy controls (female, 18/30, 60.0%; 38.4 ± 14.8 years) were enrolled. Patient and clinical characteristics and hemodynamic data are summarized in Table 1. Compared with healthy controls, ES patients had lower body mass index and systolic blood pressure and higher heart rate (all *P* < 0.05). Most ES patients were in WHO functional class II (27/45, 60.0%). Besides, we depicted in ES patients typical clinical characteristics of cyanosis and secondary erythrocytosis reflected by decreased SpO_2_ and increased Hct. We also found impaired hemodynamics manifested in elevated mPAP and PVR index (PVRI) and significant right-to-left or bidirectional shunts.

**Table 1:** Baseline characteristics of patients with ES and normal controls

	Healthy controls (*n* = 30)	ES patients (*n* = 45)	***P***-value
Clinical characteristics			
Age, years	38.4 ± 14.8	36.6 ± 11.1	0.588
Female, n, %	18 (60.0)	32 (71.1)	0.331
BSA, m^2^	1.6 ± 0.1	1.6 ± 0.2	0.987
BMI, kg/m^2^	22.1 ± 2.5	20.2 ± 3.5	**0.009**
SBP, mmHg	119 ± 8	113 ± 16	**0.031**
DBP, mmHg	73 ± 5	70 ± 11	0.145
HR, bpm	76 ± 7	89 ± 16	** < 0.001**
Hct, %	0.43 ± 0.03	0.53 ± 0.11	** < 0.001**
Plt, 10^9^/L	–	150 ± 47	–
NT-pro BNP, pg/mL	–	460 (83—1515)	–
Tnt, ng/L	–	10.9 ± 7.7	–
6MWT, m	–	424 ± 92	–
WHO class I/II/III/IV, n	–	2/27/16/0	–
SpO_2_, %	–	86 ± 5	–
Hemodynamic characteristics			
mRAP, mmHg	–	7 ± 4	–
mPAP, mmHg	–	68 ± 20	–
PAWP, mmHg	–	10 ± 4	–
Qp:Qs	–	1.0 ± 0.3	–
PVRI, Wood units × m^2^	–	26.1 ± 14.3	–
CI, L/min/m^2^		2.2 ± 0.7	–
SvO_2_, %	–	61.2 ± 9.5	–

*ES* Eisenmenger syndrome, *BSA* body surface area, *BMI* body mass index, *SBP* systolic blood pressure, *DBP* diastolic blood pressure, *HR* heart rate, *Hct* hematocrit, *NT-pro BNP* N-terminal pro-brain natriuretic peptide, *6MWT* 6-min walking test, *WHO* World Health Organization, *SpO*_*2*_ peripheral arterial oxygen saturation, *mRAP* mean right atrial pressure, *mPAP* mean pulmonary artery pressure, *PAWP* pulmonary artery wedge pressure, *Qp:Qs* pulmonary to systemic flow ratio, *PVRI* pulmonary vascular resistance index, *CI* cardiac index, *SvO*_*2*_ mixed venous oxygen saturation

Values in bold indicate P values < 0.05

### CMR morphology and function characteristics of ES and healthy controls

Significant differences in ventricular morphology and function between ES patients and healthy controls were demonstrated, including increased biventricular end-systolic volume (ESV) index (ESVI), decreased biventricular EF, decreased biventricular stroke volume index (SVI), increased LV mass index (LVMI), and increased RV ESV/LV ESV ratio (all *P* < 0.05; Table 2).

**Table 2:** CMR characteristics in patients with ES and normal controls

	Healthy controls (*n* = 30)	ES patients (*n* = 45)	*P*-value
Left ventricle, LV			
LV EDVI, mL/m^2^	78 ± 13	82 ± 30	0.512
LV ESVI, mL/m^2^	29 ± 7	39 ± 17	**0.001**
LVEF, %	62.7 ± 4.0	52.9 ± 7.9	** < 0.001**
LV mass index, g/m^2^	45 ± 8	565 ± 18	**0.002**
LV SVI, mL/m^2^	31 ± 5	39 ± 12	** < 0.001**
Right ventricle, RV			
RV EDVI, mL/m^2^	73 ± 13	122 ± 57	** < 0.001**
RV ESVI, mL/m^2^	30 ± 9	78 ± 47	** < 0.001**
RVEF, %	59 ± 7	36 ± 13	** < 0.001**
RV SVI, mL/m^2^	26 ± 4	41 ± 14	** < 0.001**
RV EDV/LV EDV	0.9 ± 0.1	1.5 ± 0.9	** < 0.001**
RV ESV/LV ESV	1.0 ± 0.2	2.3 ± 1.8	** < 0.001**
Diffuse myocardial fibrosis			
Native T1, ms	1209 ± 40	1266 ± 76	** < 0.001**
ECV, %	25.6 ± 2.2	28.6 ± 5.9	**0.004**
LGE presence, *n* (%)			
Anterior/inferior VIP	–	43 (95.6%)	–
Any myocardium*	–	16 (35.6%)	–
Septum	–	12 (26.7%)	–
RV myocardium	–	10 (22.2%)	–
LV myocardium	–	3 (6.7%)	–
RV trabeculae	–	3 (6.7%)	–
LV papillary	–	1 (2.2%)	–

*ES* Eisenmenger syndrome, *CMR* cardiovascular magnetic resonance, *LV* left ventricular, *RV* right ventricular, *EDVI* end-diastolic volume index, *ESVI* end-systolic volume index, *EF* ejection fraction, *massi* mass index, *SVI* stroke volume index, *ECV* extracellular volume, *LGE* late gadolinium enhancement, *VIP* ventricular insertion point

*Excluding anterior and inferior VIP

Values in bold indicate P values < 0.05

### Characteristics of myocardial fibrosis in patients with ES

**Fig. 2:**
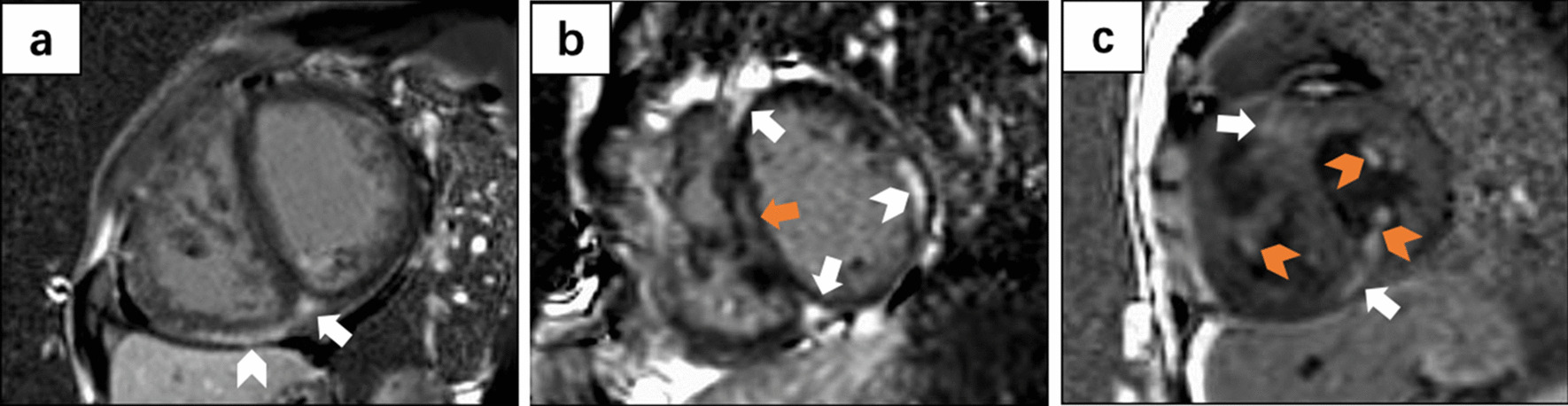
Representative examples of late gadolinium enhancement (LGE) images. **a** Inferior ventricular insertion points (VIP), white arrow) and right ventricular (RV) myocardium (white chevron); **b** Anterior/inferior VIP (white arrow), septum (orange arrow) and left ventricular (LV) myocardium (white chevron); **c** Anterior/inferior VIP (white arrow), LV papillary (orange chevron), and RV trabeculae (orange chevron). *LGE* late gadolinium enhancement, *VIP* ventricular insertion point, *LV* left ventricular, *RV* right ventricular

LGE located in anterior/inferior VIP was identified in 43 (95.6%) ES patients. Myocardial LGEs (excluding VIP) were found in 16 (35.6%) ES patients, with septal LGE in twelve (26.7%), RV myocardial LGE in ten (22.2%), LV myocardial LGE in three (6.7%), RV trabeculae LGE in three (6.7%), and LV papillary LGE in one (2.2%). Representative examples of LGE images are presented in Fig. 2. Excluding anterior/inferior VIP, the LGE^+^ and LGE^−^ subgroups had similar clinical characteristics, hemodynamics, and CMR morphology and function (Additional file 2).

In terms of diffuse myocardial fibrosis, ES patients demonstrated higher native T1 value (1266 ± 76 vs. 1209 ± 40 ms, *P* < 0.001) and ECV (28.6 ± 5.9% vs. 25.6 ± 2.2%, *P* = 0.004) than healthy controls (Table 2). When we set the upper normal native T1 limit at 1282 ms and ECV at 29.8%, 16 patients with ES (35.6%) had a native T1 value above this limit, and 17 (37.8%) had an ECV values higher than the upper limit.

**Fig. 3:**
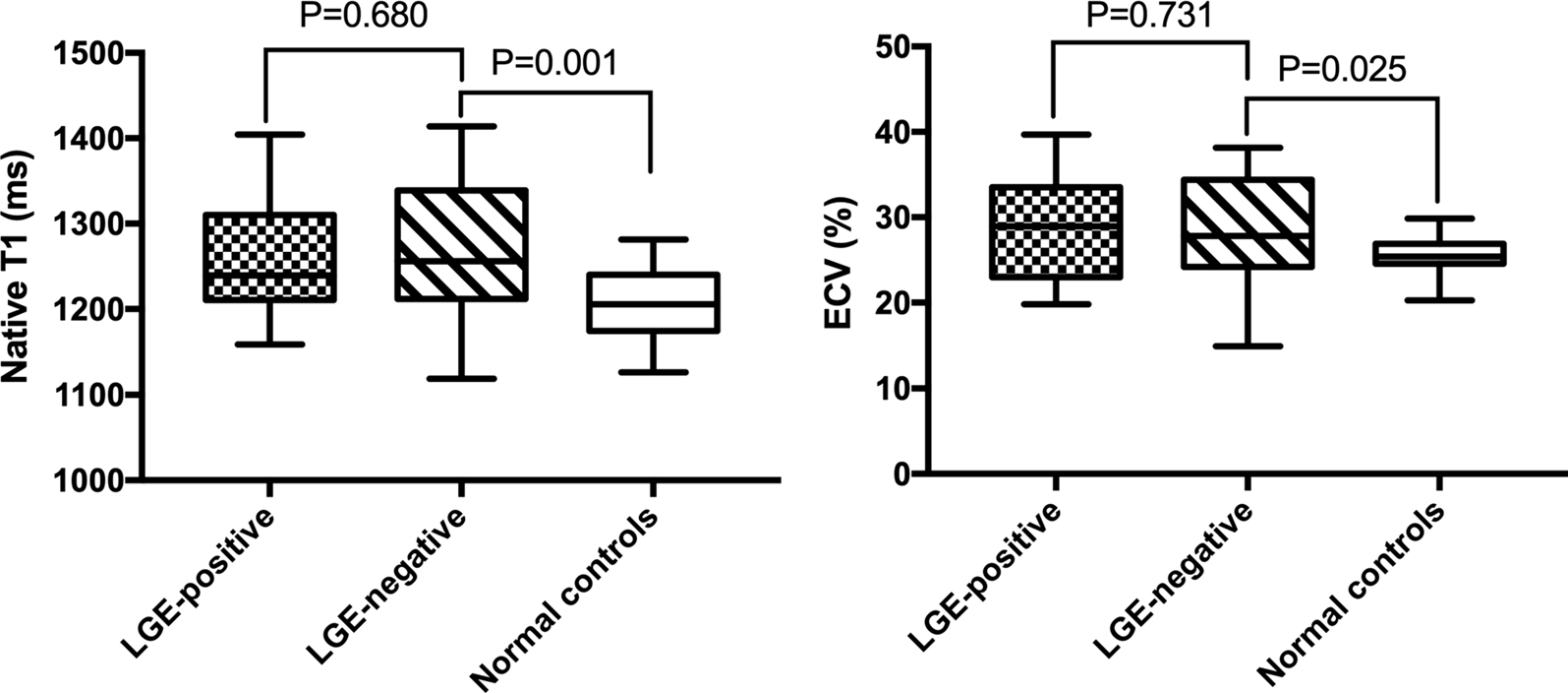
Native T1 and ECV box plots for the LGE-positive, LGE-negative, and normal control groups. *ECV* extracellular volume, *LGE* late gadolinium enhancement

Furthermore, the association between LGE and diffuse myocardial fibrosis was investigated. We found no difference in the native T1 and ECV values between the LGE^+^ and LGE^−^ subgroups (native T1, 1260 ± 18 vs. 1270 ± 15 ms, *P* = 0.68; ECV, 29.0 ± 1.5% vs. 28.3 ± 1.1%, *P* = 0.731); however, both native T1 and ECV values in ES patients in the LGE subgroup were significantly higher than in the healthy controls (both *P* < 0.05; Fig. 3).

### Correlation between diffuse ventricular fibrosis and clinical characteristics, hemodynamics, and ventricular function

**Fig. 4:**
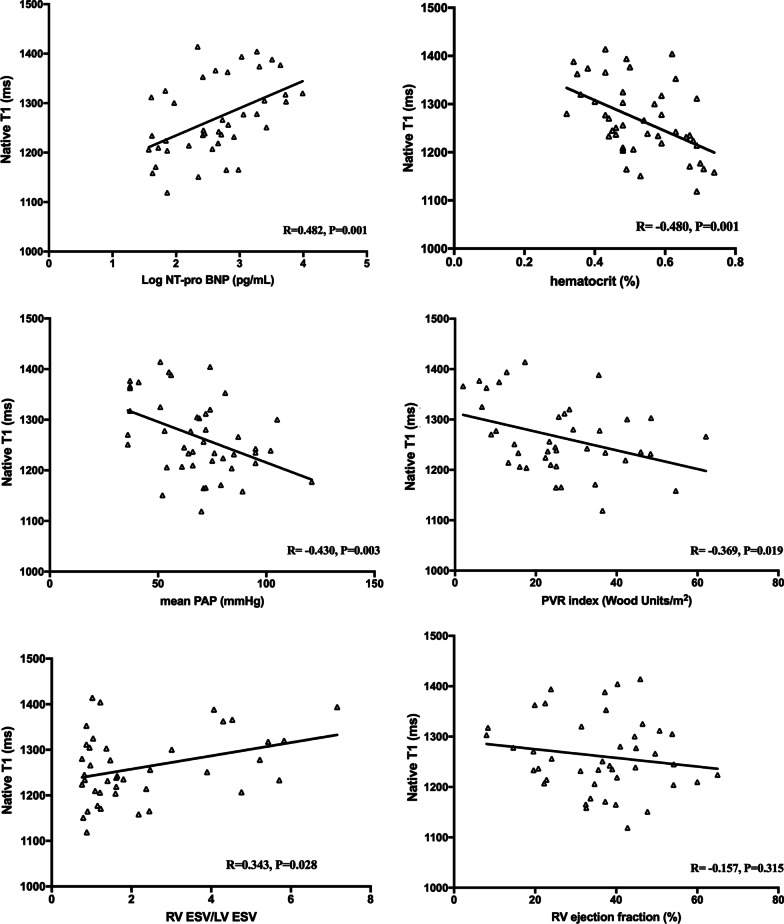
Correlations of myocardial native T1 with biomarkers, RV afterload, and RV remodeling. *NT-pro BNP* N-terminal pro-brain natriuretic peptide, *PAP* pulmonary artery pressure, *PVR* pulmonary vascular resistance, *RV* right ventricular, *LV* left ventricular, *ESV* end-systolic volume

**Fig. 5:**
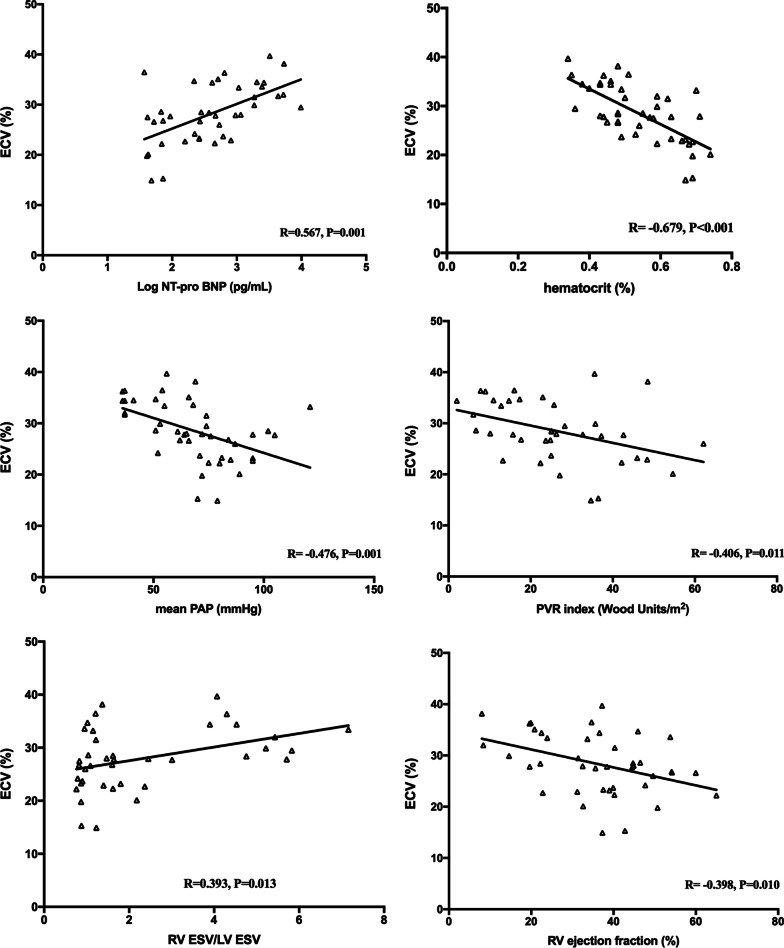
Correlation of myocardial ECV with biomarkers, RV afterload, and RV remodeling. *ECV* extracellular volume, *NT-pro BNP* N-terminal pro-brain natriuretic peptide, *PAP* pulmonary artery pressure, *PVR* pulmonary vascular resistance, *RV* right ventricular, *LV* left ventricular, *ESV* end-systolic volume

As shown in Figs. 4 and 5, compared to native T1, ECV yielded stronger correlations with serum Log NT-pro BNP (R = 0.567, *P* = 0.001), Hct (R = − 0.679, *P* < 0.001), RV afterload represented by mPAP (R = − 0.476, *P* = 0.001) and PVRI (R = − 0.406, *P* = 0.011), RV volume represented by RV ESV/LV ESV (R = 0.393, *P* = 0.013), and RV function represented by RVEF (R = − 0.398, *P* = 0.010). We found no association between native T1 and RVEF (R = 0.157, *P* = 0.315). Moreover, correlation trends between 6MWT and native T1 (R = − 0.278, *P* = 0.075) and ECV (R = − 0.299, *P* = 0.061) were observed. In the subgroup analysis, there were significant correlations or correlation trends between ECV and Log NT-pro BNP (R = 0.520, *P* = 0.003 and R = 0.527, *P* = 0.003), Hct (R = − 0.645, *P* < 0.001 and R = − 0.637, *P* < 0.001), mPAP (R = − 0.456, *P* = 0.008 and R = − 0.222, *P* = 0.238), PVRI (R = − 0.411, *P* = 0.027 and R = − 0.183, *P* = 0.372), RV/LV ESV (R = 0.390, *P* = 0.036 and R = 0.308, *P* = 0.098), and RVEF (R = − 0.493, *P* = 0.005 and R = 0.206, *P* = 0.094) in patients with intra-cardiac defects and post-tricuspid shunts, respectively.

### Prediction of risk stratification by native T1 and ECV

**Fig. 6:**
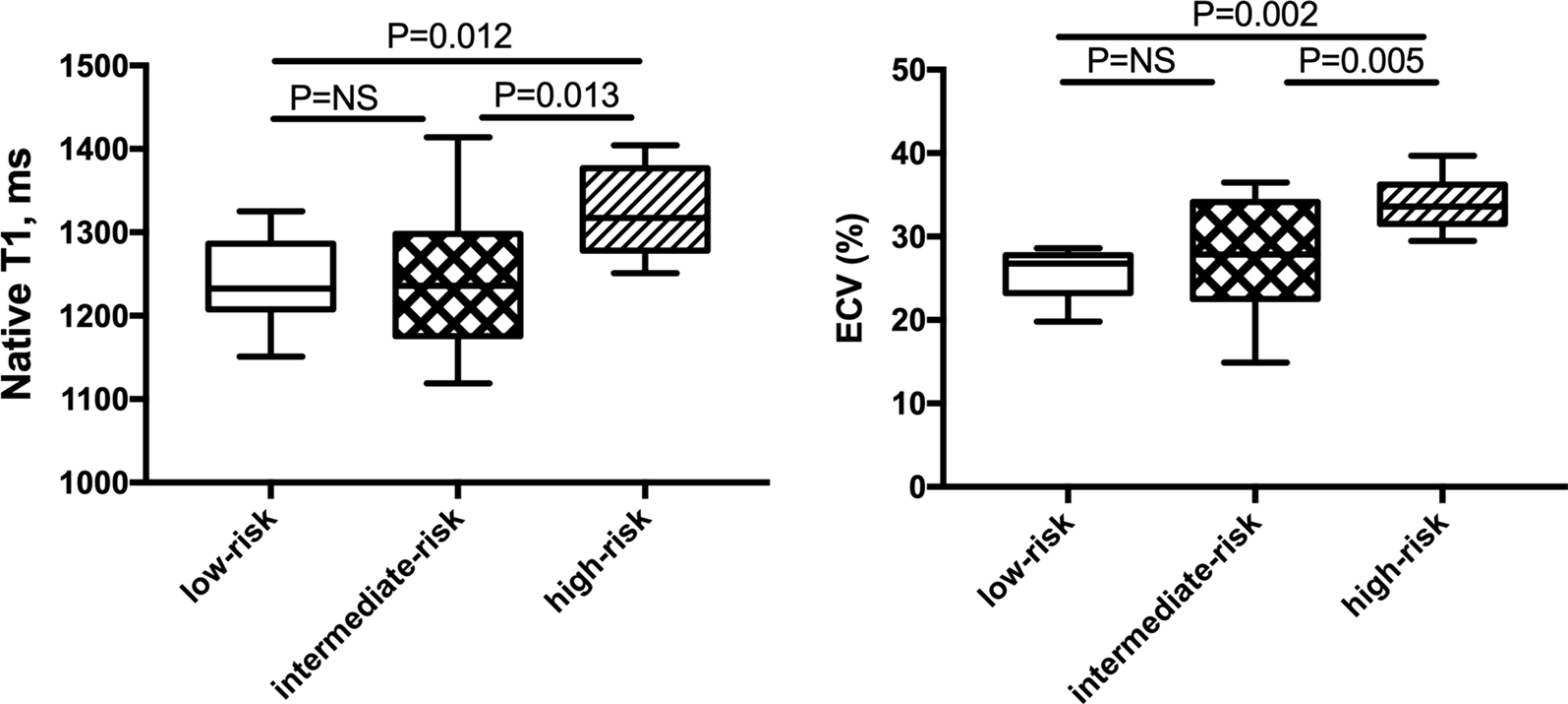
Native T1 and ECV in ES patients with various risk profiles. *ECV* extracellular volume, *ES* Eisenmenger syndrome

**Fig. 7:**
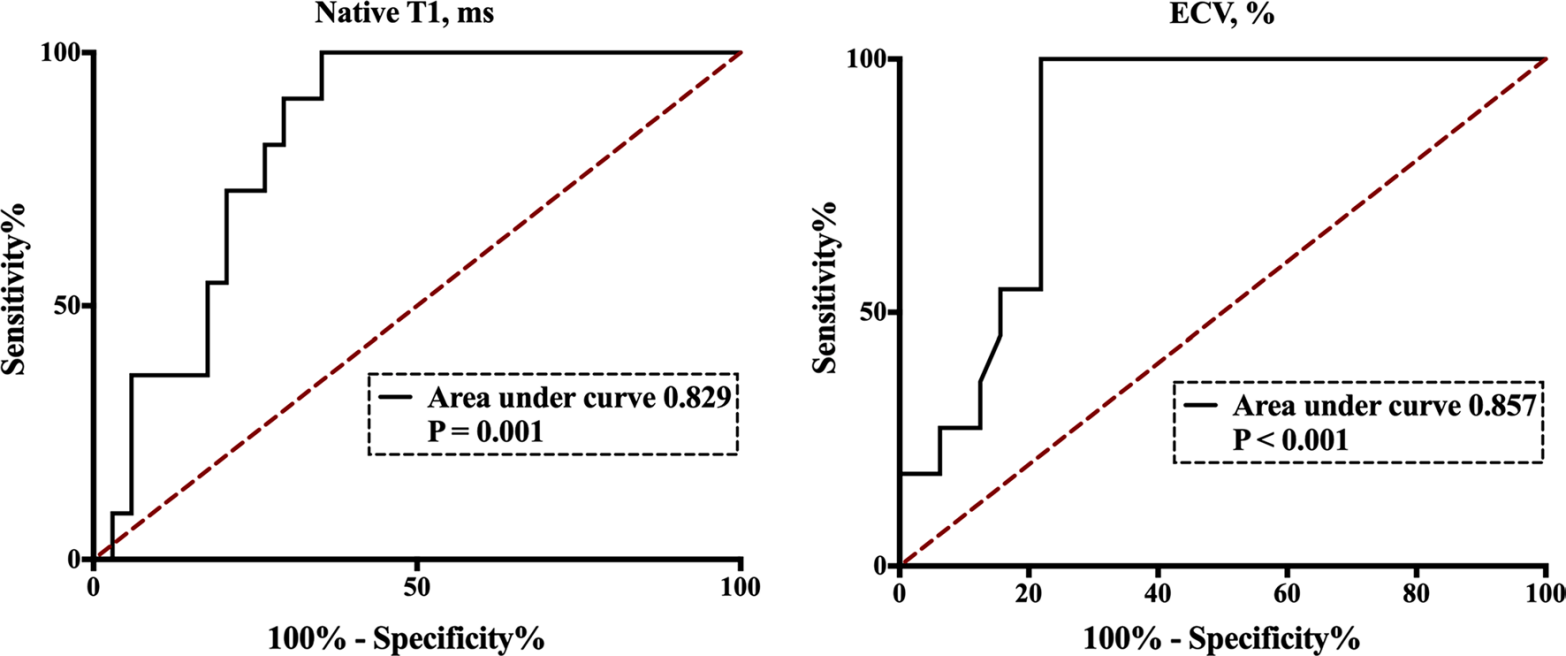
Receiver operating characteristic curves for discriminating ES patients with a high-risk profile from those with low- and intermediate-risk profiles. *ECV* extracellular volume, *ES* Eisenmenger syndrome

As shown in Fig. 6, ES patients in the high-risk group exhibited significantly higher native T1 and ECV values than patients in the low- and intermediate-risk groups (both *P* < 0.05). Furthermore, ROC curve analysis showed that an ECV threshold of 29.0% could distinguish ES patients in the high-risk group from those in the low- and intermediate-risk groups with an AUC of 0.857 (larger than for native T1; 0.829) (Fig. 7).

## Discussion

This study comprehensively evaluated myocardial fibrosis by CMR using LGE and T1 mapping. While no significant association was found between focal myocardial fibrosis as indicated by LGE and ES clinical severity, diffuse myocardial fibrosis was remarkable in ES patients and was closely associated with biomarkers of disease progression, impaired pulmonary hemodynamics, and maladaptive RV remodeling. Moreover, diffuse fibrosis performed well in identifying ES patients and a high-risk profile. Furthermore, we observed that the clinical relevance and discrimination ability of ECV were generally better than those of native T1.

CMR is the reference standard for the non-invasive imaging evaluation of myocardial fibrosis. In this study, both focal and diffuse myocardial fibrosis were detected in patients with ES using the CMR LGE and T1 mapping techniques, respectively. Broberg et al. reported that 73.0% of 30 ES patients had LGE, but both qualitative and quantitative LGE did not correlated with exercise intolerance, degree of cyanosis, ventricular size and function remodeling, and survival [12]. We have recently characterized LGE distribution in ES patients with different shunt locations [19]. However, the clinical significance of LGE and diffuse myocardial fibrosis in these patients needed to be further elucidated. In this study, LGE at the anterior/inferior VIP was very common and found in 95.6% of ES patients. Therefore, we divided patients with ES into LGE^+^ and LGE^−^ subgroups according to the presence or absence of myocardial LGE after excluding VIP. Similar with Broberg et al., we did not find the correlation between LGE and clinical disease severity or ventricular size and function. Although LGE was associated with risk stratification and the risk of cardiomyopathy-related death [28], LGE value in patients with PH has long been debated. A study evaluating 124 patients with PH found that LGE did not predict mortality [29], consistent with the findings of Freed et al. and Swift et al., who used multivariate regression analysis and found no association between LGE and additional risks in PH patients [30, 31]. Therefore, LGE in PH, particularly in the anterior/inferior VIP region, is more likely a consequence of muscle fiber disarray following an increase in septal bowing mechanical stress and interstitial space expansion due to RV hypertrophy than a sign of RV pathological decompensation.

Our study demonstrated a significantly higher diffuse myocardial fibrosis in LGE^−^ ES patients than in the healthy controls. This finding was consistent with the previous understanding that T1 mapping has been established as a substantiated and reproducible CMR technique to accurately quantify diffuse myocardial fibrosis before LGE onset [32–34]. Besides, we found no difference in diffuse myocardial fibrosis changes between those with and without LGE among patients with ES. We think it is reasonable and interpretable since we have excluded replacement fibrosis as the cause for native T1 and ECV elevation by actively avoiding contouring LGE areas in the myocardial mapping process.

Diffuse myocardial fibrosis might be the pathophysiological cause of ventricular dysfunction in ES patients. Noteworthy, only one study, published by Broberg et al. in 2010, quantified diffuse myocardial fibrosis in ten patients with cyanotic heart disease in the subgroup analysis, demonstrating a correlation between the global LV diffuse fibrosis and LV end-diastolic volume index (EDVI) and ejection fraction (EF) [13]. Nevertheless, the correlation between diffuse fibrosis and RV function has not been investigated even though right heart failure has been known as the main determinant of disease progression and poor outcomes in ES patients [2–4]. Our study provided preliminary insights into the knowledge gap in this field, showing that ES patients and various shunt locations had significantly different right heart remodeling and diffuse myocardial fibrosis [19]. These findings suggested that diffuse myocardial fibrosis might correlate with RV remodeling [19]. However, the clinical significance of diffuse myocardial fibrosis in ES patients was not investigated. In the present study, higher diffuse myocardial fibrosis was confirmed in ES patients than healthy controls and associated with increased serum NT pro-BNP level and secondary erythrocytosis, both substantial serological markers reflecting the severity of heart failure and predicting mortality in ES patients [35, 36]. Additionally, a correlation between diffuse myocardial fibrosis and increased RV afterload was observed. Previous studies have shown that diffuse myocardial fibrosis was an important byproduct of adverse ventricular loading [37]. More importantly, elevated ECV was positively correlated with maladaptive RV expansion and negatively correlated with RV systolic dysfunction. These findings supported the speculation that diffuse myocardial fibrosis had a vital role in the RV response mechanism to persistent volume and pressure overload, extending our knowledge of contributors to RV dysfunction in ES patients.

In recent years, patients with PH benefited greatly from the advent of targeted therapy [23]. The current PH guidelines recommend a risk assessment tool and risk stratification-guided targeted therapy, especially for patients with idiopathic pulmonary artery hypertension [23, 26]. The application and performance of the risk stratification strategy in patients with ES need further validation. Considering that the pathophysiology and prognosis of patients with ES are distinct from those with idiopathic pulmonary artery hypertension, increasing numbers of scholars advocate the establishment of a unique risk assessment tool for ES patients [38, 39]. In the present study, diffuse myocardial fibrosis in ES patients with a high-risk profile was significantly higher than in those with low- and intermediate-risk profiles, supporting a promising pathological marker for ES risk stratification.

A plausible explanation for the better clinical correlation and discrimination performance of ECV than native T1 mapping is that ECV could be normalized by comparing changes in T1 relaxation rates in the blood pool before and after contrast administration and was highly accurate and reproducible, while native T1 could be affected by intracellular water content, field strengths, the CMR system acquisition sequence, and individual conditions [40, 41]. All the above suggest a potential value for ECV in clinical severity assessment and a possible prognostic role in patients with ES.

### Limitations

Our study has several limitations. First, a relatively small number of subjects was recruited; however, given the low prevalence of ES, the sample size compared favorably with published studies. Second, we did not determine the predictive value of native T1 and ECV for future events (arrhythmia, death, hemoptysis, etc.) in patients with ES. Further research is warranted to determine whether alterations in diffuse ventricular fibrosis have prognostic significance and reveal whether targeting fibrosis could be a strategy to slow down the maladaptive process, prevent the development of RV failure, and allow full recovery of cardiac function in patients with ES.

## Conclusions

The marked diffuse myocardial fibrosis found in patients with ES was significantly correlated with clinical severity, RV afterload, RV size, RV systolic function, and the risk stratification profile.

## Supplementary Information

**Additional file 1. **The risk stratification approach. Low risk: at least three low-risk criteria and no high-risk criteria; Intermediate risk: definitions of low or high risk not fulfilled; High risk: at least two high-risk criteria including CI or SvO_2_. WHO, World Health Organization; 6MWT, 6-min walking distance; NT-pro BNP, N-terminal pro-brain natriuretic peptide; BNP, brain natriuretic peptide; RAP, right atrial pressure; CI, cardiac index; SvO_2_, mixed venous oxygen saturation.

**Additional file 2. **Comparison between ES patients with and without LGE. ES, Eisenmenger syndrome; BMI, body mass index; HR, heart rate; SpO_2_, peripheral arterial oxygen saturation; WHO, World Health Organization; 6MWD, 6-min walking distance; NT-pro BNP, N-terminal pro-brain natriuretic peptide; Hct, hematocrit; mPAP, mean pulmonary artery pressure; PVRi, pulmonary vascular resistance index; LV, left ventricular; RV, right ventricular; EDVi, end-diastolic volume index; ESVi, end-systolic volume index; EF, ejection fraction; massi, mass index; SVi, stroke volume index.

## Data Availability

The datasets generated and/or analyzed during the current study are available from the corresponding author on reasonable request.

## Abbreviations

6MWT: 6 Minute walk test
APVD: Anomalous pulmonary venous drainage
ASD: Atrial septal defect
AUC: Area under the curve
bSSFP: Balanced steady-state free precession
CMR: Cardiovascular magnetic resonance
CI: Cardiac index
CO: Cardiac output
ECG: Electrocardiogram
ECV: Extracellular volume fraction
EDVI: End-diastolic volume index
EF: Ejection fraction
ES: Eisenmenger syndrome
ESV: End-systolic volume
ESVI: End-systolic volume index
FA: Flip angle
FOV: Field-of-view
Hct: Hematocrit
LGE: Late gadolinium enhancement
LGE^+^: Late gadolinium enhancement positive
LGE^−^: Late gadolinium enhancement negative
LV: Left ventricle/left ventricular
LVMI: Left ventricular mass index
MOLLI: Motion-corrected modified Look-Locker inversion recovery
mPAP: Mean pulmonary artery pressure
mRAP: Mean right atrial pressure
NT-pro BNP: N-terminal pro-brain natriuretic peptide
PAWP: Pulmonary artery wedge pressure
PDA: Patent ductus arteriosus
PH: Pulmonary hypertension
PVR: Pulmonary vascular resistance
PVRI: Pulmonary vascular resistance index
Qp:Qs: Pulmonary to systemic flow ratio
RHC: Right heart catheterization
ROC: Receiver operating characteristic
ROI: Region of interest
RV: Right ventricle/right ventricular
RVEF: Right ventricular ejection fraction
SpO_2_: Peripheral arterial oxygen saturation
SvO_2_: Mixed venous oxygen saturation
SVI: Stroke volume index
TE: Echo time
TR: Repetition time
TI: Inversion time
VIP: Ventricular insertion point
VSD: Ventricular septal defect
VIP: Ventricular insertion point
WHO: World Health Organization
