# Supplementary material for: Detection and evaluation of myocardial fibrosis in Eisenmenger syndrome using cardiovascular magnetic resonance late gadolinium enhancement and T1 mapping
Source: J Cardiovasc Magn Reson. 2022 Nov 21;24:60. doi: 10.1186/s12968-022-00880-2 (PMC9677680; doi:10.1186/s12968-022-00880-2)
Supplement: Supplementary file 1 — Additional file 1. The risk stratification approach. Low risk: at least three low-risk criteria and no high-risk criteria; Intermediate risk: definitions of low or high risk not fulfilled; High risk: at least two high-risk criteria including CI or SvO2. WHO, World Health Organization; 6MWT, 6-min walking distance; NT-pro BNP, N-terminal pro-brain natriuretic peptide; BNP, brain natriuretic peptide; RAP, right atrial pressure; CI, cardiac index; SvO2, mixed venous oxygen saturation. [file 12968_2022_880_MOESM1_ESM.docx]

**Additional file 1**. The risk stratification approach.

|  | **Prognosis factors** | **Low risk** | **Intermediate risk** | **High risk** |
| --- | --- | --- | --- | --- |
| Criteria A | WHO functional class | I/II | III | IV |
| Criteria B | 6MWD | > 440 m | 165 - 440 m | < 165 m |
| Criteria C | NT-proBNP/BNP or RAP (Take the worse indicator of the two) | NT-pro BNP < 300ng/l, BNP < 50ng/l or RAP < 8 mmHg | NT-pro BNP 300 - 1400ng/l, BNP 50 - 300ng/l or RAP 8 – 14 mmHg | NT-pro BNP > 1400ng/l, BNP > 300ng/l or RAP > 14 mmHg |
| Criteria D | CI or SvO_2_ (Take the worse indicator of the two) | CI ≥ 2.5 l/min/m^2^ or SvO_2_ > 65% | CI 2.0 – 2.4 l/min/m^2^ or SvO_2_ 60 – 65% | CI < 2.0 l/min/m^2^ or SvO_2_ < 60% |

WHO, World Health Organization; 6MWD, 6-minute walking distance; NT-pro BNP, N-terminal pro-brain natriuretic peptide; BNP, brain natriuretic peptide; RAP, right atrial pressure; CI, cardiac index; SvO_2_, mixed venous oxygen saturation.

Low risk: at least three low-risk criteria and no high-risk criteria.

Intermediate risk: definitions of low or high risk not fulfilled.

High risk: at least two high-risk criteria including CI or SvO_2_.
