# Supplementary material for: Detection and evaluation of myocardial fibrosis in Eisenmenger syndrome using cardiovascular magnetic resonance late gadolinium enhancement and T1 mapping
Source: J Cardiovasc Magn Reson. 2022 Nov 21;24:60. doi: 10.1186/s12968-022-00880-2 (PMC9677680; doi:10.1186/s12968-022-00880-2)
Supplement: Supplementary file 2 — Additional file 2. Comparison between ES patients with and without LGE. ES, Eisenmenger syndrome; BMI, body mass index; HR, heart rate; SpO2, peripheral arterial oxygen saturation; WHO, World Health Organization; 6MWD, 6-min walking distance; NT-pro BNP, N-terminal pro-brain natriuretic peptide; Hct, hematocrit; mPAP, mean pulmonary artery pressure; PVRi, pulmonary vascular resistance index; LV, left ventricular; RV, right ventricular; EDVi, end-diastolic volume index; ESVi, end-systolic volume index; EF, ejection fraction; massi, mass index; SVi, stroke volume index. [file 12968_2022_880_MOESM2_ESM.docx]

**Additional file 2**. Comparison between ES patients with and without LGE.

|  | **LGE-negative**  **(n = 29)** | **LGE-positive**  **(n = 16)** | **P-value** |
| --- | --- | --- | --- |
| Age, years | 34.8 ± 12.3 | 39.9 ± 8.0 | 0.142 |
| Female, n, % | 22 (75.9) | 10 (62.5) | 0.494 |
| BMI, kg/m^2^ | 20.3 ± 3.4 | 20.0 ± 3.9 | 0.787 |
| SpO_2_, % | 86.2 ± 4.7 | 86.8 ± 5.2 | 0.695 |
| WHO class I/II/III/IV, n | 2/14/13/0 | 0/13/3/0 | 0.084 |
| 6MWD, m | 417.1 ± 92.8 | 439.0 ± 93.3 | 0.477 |
| Log NT-pro BNP, pg/mL | 2.6 ± 0.7 | 2.7 ± 0.7 | 0.548 |
| mPAP, mmHg | 65.4 ± 19.8 | 73.9 ± 20.3 | 0.180 |
| PVRi, Wood units/m^2^ | 23.8 ± 14.5 | 31.0 ± 12.9 | 0.133 |
| LV EDVi, mL/m^2^ | 80.1 ± 28.7 | 83.8 ± 33.8 | 0.713 |
| LV ESVi, mL/m^2^ | 38.4 ± 17.4 | 41.0 ± 16.2 | 0.640 |
| LV EF, % | 53.4 ± 7.8 | 52.1 ± 8.4 | 0.610 |
| LV massi, g/m^2^ | 51.6 ± 17.7 | 62.8 ± 15.4 | 0.055 |
| LV SVi, mL/m^2^ | 40.0 ± 11.5 | 36.1 ± 13.0 | 0.344 |
| RV EDVi, mL/m^2^ | 118.5 ± 55.2 | 129.0 ± 62.4 | 0.578 |
| RV ESVi, mL/m^2^ | 80.2 ± 51.3 | 73.5 ± 37.5 | 0.668 |
| RV EF, % | 36.0 ± 13.1 | 36.1 ± 14.1 | 0.983 |
| RV SVi, mL/m^2^ | 39.6 ± 13.6 | 42.9 ± 15.0 | 0.455 |
| RV EDV/LV EDV | 1.6 ± 1.0 | 1.5 ± 0.9 | 0.744 |
| RV ESV/LV ESV | 2.4 ± 1.9 | 2.1 ± 1.5 | 0.678 |

ES, Eisenmenger syndrome; BMI, body mass index; HR, heart rate; SpO_2_, peripheral arterial oxygen saturation; WHO, World Health Organization; 6MWD, 6-minute walking distance; NT-pro BNP, N-terminal pro-brain natriuretic peptide; Hct, hematocrit; mPAP, mean pulmonary artery pressure; PVRi, pulmonary vascular resistance index; LV, left ventricular; RV, right ventricular; EDVi, end-diastolic volume index; ESVi, end-systolic volume index; EF, ejection fraction; massi, mass index; SVi, stroke volume index.
